# Effect of a lime-based bedding conditioner on physical-chemical characteristics and microbiological counts of recycled manure solids

**DOI:** 10.3389/fvets.2024.1408798

**Published:** 2024-07-15

**Authors:** Gustavo Freu, Sara Fusar Poli, Valentina Monistero, Filippo Biscarini, Nicola Rota, Delower Hossain, Claudia Gusmara, Laura Musa, Gloria Gioia, Lorenzo Leso, Marcos Veiga dos Santos, Paolo Moroni, Maria Filippa Addis, Valerio Bronzo

**Affiliations:** ^1^Department of Animal Nutrition and Production, School of Veterinary Medicine and Animal Science, University of São Paulo (USP), Pirassununga, São Paulo, Brazil; ^2^Department of Veterinary Medicine and Animal Sciences—DIVAS, University of Milan, Lodi, Italy; ^3^Laboratorio di Malattie Infettive degli Animali—MiLab, University of Milan, Lodi, Italy; ^4^National Research Council, Institute of Agricultural Biology and Biotechnology (CNR-IBBA), Milan, Italy; ^5^Agribovis s.r.l., Meda, Italy; ^6^Department of Medicine and Public Health, Faculty of Animal Science and Veterinary Medicine, Sher-e-Bangla Agricultural University, Dhaka, Bangladesh; ^7^Quality Milk Production Services, Animal Health Diagnostic Center, Cornell University, Ithaca, NY, United States; ^8^Department of Agricultural, Food, and Forest Sciences—SAAF, University of Palermo, Palermo, Italy

**Keywords:** anaerobically digested manure solids, separated raw manure solids, bedding conditioner, bedding management, microbiological counts

## Abstract

Bedding materials are aimed at providing a safe and comfortable resting environment for cows. Control of pathogen proliferation in these substrates is crucial to prevent intramammary infections in dairy cows, as these can significantly impact milk quality, cow health, and farm productivity. This is particularly relevant in the case of organic bedding substrates, including manure-derived materials. This study aimed to evaluate the *in vitro* effect of a lime-based conditioner (LBC), composed of CaCO_3_MgCO_3_ and ${\text{Ca(OH)}}_{2}^{*}$Mg(OH)_2_, at increasing concentrations on the physical-chemical characteristics and bacterial counts of untreated anaerobically digested manure solids (ADMS) and separated raw manure solids (SRMS). Unused ADMS and SRMS were evaluated at four LBC weight-based concentrations: 0 (as untreated control), 10, 15, and 20% of LBC inclusion. The bedding materials were assessed immediately after LBC addition (0 h) and after 24, 72, and 168 h of storage at 28°C. The dry matter content (DM), and pH were measured for all the time points. Standard microbiological methods were used to assess total bacterial counts (TBC), other Gram-negative bacteria, coliforms, *Escherichia coli*, and streptococci and streptococci-like organism (SSLO). It was observed a linear increase in both DM and pH with increasing concentrations of LBC. Specifically, for each percentage unit increase of LBC, the DM of ADMS and SRMS increased by 0.73 and 0.71%, respectively. Similarly, for each percentage unit of LBC, the pH of ADMS and SRMS increased by 0.15 and 0.19, respectively. Conversely, a linear decrease in TBC, Gram-negative bacteria, coliforms, *E. coli*, and SSLO was observed with increasing concentrations of the LBC. Manure-derived materials without the inclusion of the LBC had bacterial counts that tended to remain high or increase over time. Otherwise, bedding materials with LBC application had reduced bacterial counts. Based on the results of the present study, it was observed that the higher the concentration of LBC, the more significant the reduction of bacterial counts. Specifically, bacterial recovery was lower when higher concentrations of LBC were applied. Our findings underscore the potential of LBC in effectively controlling environmental bacteria and improving the physical-chemical characteristics of manure-derived bedding materials to improve cow health and welfare.

## 1 Introduction

Several substrates can be used as bedding in dairy farms; however regardless of the substrate, a comfortable and safe resting area for cows is essential. Organic materials derived from manure solids have gained attention due to their soft, non-abrasive characteristics, which have a positive impact on animal welfare (1–3). These substrates, also known as recycled manure solids (RMS), have been increasingly used in North America over the last decade (3, 4), and are gaining usage in Europe (1). Typically, these solids are obtained by screwing raw (undigested) slurry through a screw press to separate solids from liquid and should achieve a dry matter (DM) content of at least 34% (5). Despite the benefits, RMS are known to carry a higher bacterial load than other bedding substrates (5) and have been associated with increased mastitis risk (6). Therefore, strategies to manage the bacterial counts within RMS to reduce udder exposure need to be investigated since this bedding could have an impact on cow health (7).

According to the European Union's EC Regulation 1069/2009, livestock manure is Category 2 Animal By-product and can only be used as bedding for dairy cows (as a “technical product”) under conditions that do not pose a risk to the to human or animal health (8). Bedding conditioners can be used by farmers to control pathogen population in bedding substrates. Most conditioners alter the pH of bedding materials (e.g., acidic or alkaline conditioners) suppressing microbial growth (1, 9). Hydrated lime is a common alkaline compound formed by calcium hydroxide, which can raise the pH of the materials to which it is added to levels above 12 (10), creating a substrate inhospitable to bacterial growth.

Previous studies evaluated the effect of hydrated lime on microbial populations in different bedding materials (9–11), and reduction in bacterial counts was observed in sawdust (12), and RMS treated with 10% of hydrated lime (11). Furthermore, adding 0.5 kg hydrated lime every 48 h on free-stall mattresses reduced coliforms, and *Streptococcus* spp. counts (9). Although the efficacy of bedding conditioners is well-documented, their effects are usually short-lived, typically lasting between 24 and 48 h (13). In a US study, the addition of lime to sawdust reduced bacterial counts, but the effect only lasted for 1 d (11). Further, adding lime daily to the top layer of straw failed to raise the pH to those inhibitory levels at which *E. coli* and *Streptococcus uberis* do not survive (14).

Scientific evidence for optimum bedding management remains limited and occasionally conflicting (1). To our knowledge, no studies have been conducted to assess the impact of varying lime-based conditioner (LBC) concentrations on microbial populations of organic bedding substrates. Such investigations can offer the first valuable insights for veterinarians and farmers seeking effective bedding management and mastitis control from a laboratory point of view and a starting point on the base of the results for farms trial. To address this scientific gap, this study aimed to evaluate the *in vitro* effect of a commercial LBC at increasing concentrations on the physical-chemical characteristics and bacterial counts of anaerobically digested manure solids (ADMS) and separated raw manure solids (SRMS), as well as the temporal effect of the LBC.

## 2 Materials and methods

### 2.1 Bedding substrates collection and study protocols

From February/23 to May/23, unused ADMS and SRMS were collected from on-farm storage areas of two commercial dairy farms where 700 and 650 Holstein cows respectively were housed in free stall systems. Bedding sampling was performed according to Godden et al. (15). Briefly, ~5 kg of each bedding substrate was collected from the bedding storage area into a sterile plastic bag by a trained personal from Agribovis wearing clean gloves (15). None of the study herds were using bedding conditioner or RMS as bedding at the time of sample collection.

The ADMS and SRMS were collected 24 h after on-farm production from 5 random locations within the storage area. After being taken out of the biodigester (~40 d at 38°C), ADMS was sent to the manure separator (SEPCOM Bedding, WAMGROUP, Cavezzo, Italy), followed by sample collection. SRMS were sampled directly from the storage area of the separator (SEPCOM Bedding, WAMGROUP, Cavezzo, Italy). Subsequently, the samples were immediately transported (<1 h) in an isothermal box (~4°C) to the Animal Infectious Disease Laboratory (MiLab) of the University of Milan for treatment allocation and further analysis.

### 2.2 Treatments

Bedding substrates (ADMS, and SRMS) were evaluated in untreated control sample (0%, no LBC addition) and at three concentrations of LBC (VF10^®^, Unicalce, Lecco, Italy), which included: 10, 15, and 20% of product inclusion (% by wet weight). The LBC used in this study was composed of calcium carbonate, magnesium, and semi-hydrated lime [CaCO_3_MgCO_3_ and ${\text{Ca(OH)}}_{2}^{*}$Mg(OH)_2_]. The product inclusion was defined based on previous reports (11, 16), and on the company recommendation based on an internal study. Starting from 350 g of each substrate sample, the following allocation scheme was adopted: 0% (350 g of untreated sample), 10% (35 g of LBC + 315 g of sample), 15% (52.5 g of LBC + 297.5 g of sample), and 20% (70 g of LBC + 280 g of sample). The LBC was added to the bedding substrates as soon as they arrived at the laboratory and was manually mixed to ensure homogeneity. Subsequently, each substrate sample was weighed in duplicate into a sterile container, and additional aliquots were prepared for DM analysis.

### 2.3 Dry matter content, pH, and microbiological analyses

The initial DM, pH, and microbiological analysis were carried out immediately after LBC application (0 h). Additional DM, pH, and microbiological analysis were performed after 24, 72, and 168 h of sample storage at 28°C, to simulate environmental conditions and to provide a standardized baseline to compare treatments and to assess their effects under controlled conditions.

For DM estimation, bedding samples (~10.0 g) were weighed in duplicate (initial weigh) and placed into an oven at 100 ± 10°C for 24 h. After drying, samples were reweighed (final weight), with precision of two decimals places (17).

At each time point, 25 g of each bedding sample were weighted in duplicate by taking small sub-samples from at least 3 random locations within the mixed sample and were transferred to two different filter stomacher bags, where 225 mL of physiological salt solution (PSS; NaCl 0.9%) was added to each of them. The suspension was homogenized for 90 s at 8 strokes/s using a BagMixer 400 W (Interscience, Puycapel, France). The two replicates were combined into a flask, from which, a sub-sample (~50 mL) was separated for pH measurement. The pH was accessed using a pH meter (pH 50 Violab, Carpi, Italy) as previously reported (17).

For microbiological analysis, two aliquots of 5 mL from the aforementioned flask were transferred into two sterile dilution tubes to create a duplicate 10^−1^ dilution. Serial dilutions up to 10^−8^ were prepared by vortexing the sample and transferring 0.5 mL into a new tube containing 4.5 mL of PSS. Subsequently, 100 μL of each dilution was streaked in triplicate onto Chromogenic (CHR) agar plates (CHROMagar^TM^ ECC, Paris, France; from 10^−1^ to 10^−5^), and Edwards (EDW) modified medium plates (Oxoid, Basingstoke, UK; from 10^−1^ to 10^−6^) supplemented with 5.0% bovine blood. The plates were incubated at 37°C. After 24 h of incubation, CHR plates allowed to distinguish Gram-negative bacteria into *E. coli*, coliforms, and other Gram-negative. After 48 h, streptococci and streptococci-like organism (SSLO) counts were performed from EDW plates. Because phenotypic and biochemical identification methods can be inaccurate and unreliable for species within the *Streptococcus* group (e.g., *Aerococcus* spp., *Enterococcus* spp., and *Lactococcus* spp.), for EDW plates, blue/black colonies were counted together and reported as SSLOs as previously reported (18). To determine the total bacterial count (TBC), 1,000 μL of the inoculum was added in triplicate (from 10^−3^ to 10^−8^) at the center of each plate. Approximately 15 mL of molten plate count agar (Microbiol Diagnostic, Cagliari, Italy) was added. Plates were manually homogenized, and read after incubation at 37°C for 72 h.

Each culture medium was prepared according to the manufacturer's recommendations. Bacterial counting was performed manually, and the results were normalized to the DM content as previously described (17). The results of microbial counts were expressed as log_10_ cfu/g.

### 2.4 Statistical analysis

The available dataset had a multilevel hierarchical organization with a nested structure. The analysis was conducted separately for the ADMS and SRMS bedding substrates. For each bedding type, both a within-timepoint and across-timepoint analysis were carried out. For the within-timepoint analysis, the data were analyzed within each timepoint (0, 24, 72, and 168 h after LBC addition) using the following model:


(1) $$\begin{array}{l} y_{ij}=\beta_{0}\;+\beta_{1}^{*}treatment_{i}\;+e_{ij} \end{array}$$

where *y*_*ij*_ is the value for pH, DM or the bacterial counts (log_10_ cfu/g) for sample *i* and LBC dose *j*; β_0_ is the intercept; β_1_ is the coefficient of the treatment effect; *treatment*_*i*_ is the effect of incremental doses of LBC (between 0 and 20%); *e*_*ij*_ are the model residuals.

For the across-timepoint analysis, all data (for ADMS and SRMS separately) were analyzed jointly with the following model (Equation 2):


(2) $$\begin{array}{l} y_{ijk}=\beta_{0}\;+\beta_{1}^{*}timepoint_{i}+\beta_{2}^{*}treatment_{j}+e_{ijk} \end{array}$$

where all terms were as in Equation 1 with the addition of *timepoint*_*i*_ that is effect of the number of hours from the LBC application (four classes).

Data collected and laboratory results were stored in Excel spreadsheets (19). Data handling and statistical analysis were performed using the R environment for statistical computing (20), specifically using the package dplyr (21). A significance level of *P* < 0.05 was considered.

## 3 Results

### 3.1 Dry matter content and pH

DM content and pH results (mean ± SD) are shown in Table 1. A linear increase in DM content according to the LBC concentration was observed for both ADMS and SRMS (*P* < 0.001; Table 2). No differences were observed in the DM content of ADMS over time (*P* ≥ 0.12), while for SRMS an increasing pattern in DM content over time was observed (*P* ≤ 0.009; Table 3). However, for both substrates, bedding treatment had a positive effect on DM content, i.e., for each % unit increase in LBC concentration corresponded to a 0.73% DM increase for ADMS (*P* < 0.001) and 0.72% for SRMS (*P* < 0.001; Table 3). There was no interaction effect for DM between LBC concentrations and time of evaluation (*P* = 0.258).

**Table 1:** Descriptive statistics of dry matter (DM) and pH results (mean ± SD^a^) of anaerobically digested manure solids and separated raw manure solids treated with increasing concentrations of lime-based conditioner according to the evaluated lab-times.

**Variable**	**Time-points (h)^b^**	**ADMS** ^**c**^				**SRMS** ^**d**^			
		**0%^e^**	**10%^f^**	**15%^g^**	**20%^h^**	**0%**	**10%**	**15%**	**20%**
DM	0	32.49 ± 0.02	40.68 ± 0.44	43.87 ± 0.20	47.40 ± 0.44	32.14 ± 0.28	39.15 ± 0.18	42.16 ± 0.36	45.71 ± 0.28
	24	32.72 ± 0.16	40.82 ± 0.43	43.85 ± 0.14	47.07 ± 0.13	32.67 ± 0.51	39.80 ± 0.18	42.69 ± 0.22	46.52 ± 0.69
	72	33.18 ± 0.19	41.18 ± 0.54	44.62 ± 0.43	47.58 ± 0.26	32.69 ± 0.51	39.97 ± 0.61	42.93 ± 0.26	47.26 ± 0.26
	168	33.18 ± 0.51	41.26 ± 1.08	43.68 ± 2.82	48.16 ± 0.87	32.55 ± 0.72	39.83 ± 0.41	44.66 ± 0.26	48.18 ± 0.51
pH	0	8.66 ± 0.00	10.95 ± 0.00	11.71 ± 0.00	12.02 ± 0.00	8.54 ± 0.03	11.35 ± 0.02	11.97 ± 0.02	12.22 ± 0.01
	24	8.71 ± 0.01	10.14 ± 0.01	10.74 ± 0.03	11.64 ± 0.02	7.97 ± 0.01	10.31 ± 0.01	11.09 ± 0.01	11.93 ± 0.01
	72	8.51 ± 0.01	10.10 ± 0.02	10.51 ± 0.01	11.46 ± 0.04	7.69 ± 0.01	9.29 ± 0.02	10.47 ± 0.02	11.72 ± 0.03
	168	8.42 ± 0.00	9.25 ± 0.00	10.38 ± 0.00	11.38 ± 0.00	7.81 ± 0.01	9.01 ± 0.00	10.19 ± 0.02	11.42 ± 0.01

^a^Standard deviation.

^b^Sample processed immediately (0 h), and after 24, 72, and 168 h of storage at 28°C.

^c^Anaerobically digested manure solids.

^d^Separated raw manure solids.

^e^0%: untreated control sample.

^f^10%: sample treated with 10% of lime-based conditioner.

^g^15%: sample treated with 15% of lime-based conditioner.

^h^20%: sample treated with 20% of lime-based conditioner.

**Table 2:** Within-timepoint models^a^ for dry matter (DM) content (%) and pH of anaerobically digested manure solids and separated raw manure solids treated with lime-based conditioner.

**Variable**	**Time-points (h)^b^**	**Term^c^**	**ADMS** ^**d**^				**SRMS** ^**e**^			
			**Estimate**	**SE^f^**	**Statistic**	***P*-value**	**Estimate**	**SE**	**Statistic**	***P*-value**
DM	0	Treatment	0.745	0.017	43.255	<0.001	0.675	0.011	59.324	<0.001
	24		0.718	0.018	39.023	<0.001	0.686	0.018	38.207	<0.001
	72		0.726	0.020	35.616	<0.001	0.717	0.02	36.708	<0.001
	168		0.733	0.059	12.439	<0.001	0.791	0.022	35.518	<0.001
pH	0	Treatment	0.174	0.012	14.325	<0.001	0.190	0.018	10.524	<0.001
	24		0.143	0.003	49.067	<0.001	0.199	0.006	31.808	<0.001
	72		0.143	0.005	29.433	<0.001	0.199	0.007	26.687	<0.001
	168		0.147	0.011	12.948	<0.001	0.179	0.011	16.556	<0.001

^a^Within-timepoint model assessed the effects of incremental lime-based conditioner doses (0–20%) on DM and pH, at each timepoint (0, 24, 72, and 168 h).

^b^Sample processed immediately (0 h), and after 24, 72, and 168 h of storage at 28°C.

^c^Treatment: untreated control sample, and sample treated with 10, 15, and 20% of lime-based conditioner.

^d^Anaerobically digested manure solids.

^e^Separated raw manure solids.

^f^Standard error.

**Table 3:** Across-timepoint models^a^ for dry matter (DM) content and pH of anaerobically digested manure solids and separated raw manure solids treated with lime-based conditioner.

**Variable**	**Term^b^**	**ADMS** ^**c**^				**SRMS** ^**d**^			
		**Estimate**	**SE^e^**	**Statistic**	***P*-value**	**Estimate**	**SE**	**Statistic**	***P*-value**
DM	24 h	0.006	0.341	0.017	0.987	0.634	0.234	2.708	0.009
	72 h	0.535	0.341	1.570	0.124	0.925	0.234	3.950	<0.001
	168 h	0.462	0.341	1.357	0.182	1.515	0.234	6.470	<0.001
	Treatment	0.731	0.016	44.866	<0.001	0.717	0.011	64.034	<0.001
pH	24 h	−0.529	0.097	−5.435	<0.001	−0.696	0.120	−5.809	<0.001
	72 h	−0.691	0.097	−7.095	<0.001	−1.227	0.120	−10.241	<0.001
	168 h	−0.978	0.097	−10.039	<0.001	−1.411	0.120	−11.779	<0.001
	Treatment	0.152	0.005	32.612	<0.001	0.192	0.006	33.458	<0.001

^a^For the across-timepoint analysis, all data (for ADMS and SRMS separately) were analyzed jointly, considering the effects of incremental lime-based conditioner doses (0–20%) on DM and pH, and including in the model the effect of the number of hours of lime-based conditioner application.

^b^Sample processed after 24, 72, and 168 h of storage at 28°C, respectively; Treatment: untreated control sample, and sample treated with 10, 15, and 20% of lime-based conditioner.

^c^Anaerobically digested manure solids.

^d^Separated raw manure solids.

^e^Standard error.

The pH results showed a linear increase in pH values with increasing LBC concentration for both substrates (*P* < 0.001; Table 2). As expected, a decreasing pattern in pH over time was observed for both substrates (*P* < 0.001; Table 3). Similar to DM results, for each % unit of LBC inclusion pH increased by 0.15 for ADMS (*P* < 0.001) and 0.19 for SRMS (*P* < 0.001; Table 3). There was no interaction effect for pH between LBC concentration and time of evaluation (*P* = 0.542).

### 3.2 Microbiological counts

The microbiological results of ADMS and SRMS treated with varying concentrations of LBC across evaluated time-points are shown in Table 4. Regarding TBC, the inclusion of LBC resulted in lower TBC in both ADMS and SRMS compared to untreated samples throughout the study period (Table 4). Regardless of the evaluation time, a linear decrease in TBC was observed with increasing LBC concentrations (*P* < 0.001; Table 5). Over time, a consistently increasing pattern in TBC was observed for both substrates, with particularly significant increases observed for ADMS at 72 and 168 h (*P* < 0.001) and for SRMS at 168 h (*P* = 0.004; Table 6). Each % unit of LBC inclusion corresponded to a reduction of TBC by 0.16 log_10_ cfu/g for ADMS (*P* < 0.001) and 0.12 log_10_ cfu/g for SRMS (*P* < 0.001; Table 6).

**Table 4:** Descriptive statistics of microbiological count results (mean ± SD^a^ log_10_ cfu/g) of anaerobically digested manure solids and separated raw manure solids treated with increasing concentrations of lime-based conditioner according to the evaluated lab-times.

**Bacterial group**	**Time-points (h)^b^**	**ADMS** ^**c**^				**SRMS** ^**d**^			
		**0%^e^**	**10%^f^**	**15%^g^**	**20%^h^**	**0%**	**10%**	**15%**	**20%**
TBC^i^	0	9.17 ± 0.08	8.25 ± 0.01	7.31 ± 0.08	6.50 ± 0.10	9.58 ± 0.03	9.20 ± 0.04	8.90 ± 0.11	8.68 ± 0.10
	24	10.12 ± 0.08	7.76 ± 0.09	7.30 ± 0.16	6.65 ± 0.10	10.41 ± 0.04	9.27 ± 0.05	8.16 ± 0.06	7.34 ± 0.09
	72	10.34 ± 0.05	9.24 ± 0.18	7.84 ± 0.08	6.82 ± 0.07	10.43 ± 0.06	9.92 ± 0.08	8.85 ± 0.08	7.72 ± 0.08
	168	9.97 ± 0.03	9.96 ± 0.10	7.95 ± 0.11	7.03 ± 0.02	10.67 ± 0.06	10.05 ± 0.08	9.26 ± 0.05	7.82 ± 0.13
Total Gram-negative	0	5.70 ± 0.06	0.88 ± 1.37	0.39 ± 0.96	0.00 ± 0.00	5.16 ±0.10	0.80 ± 1.24	0.00 ± 0.00	0.00 ± 0.00
	24	6.04 ± 0.12	0.00 ± 0.00	0.00 ± 0.00	0.00 ± 0.00	6.95 ± 0.07	2.36 ± 1.16	0.00 ± 0.00	0.00 ± 0.00
	72	7.17 ± 0.04	1.69 ± 1.32	0.00 ± 0.00	0.00 ± 0.00	5.65 ± 0.06	0.00 ± 0.00	0.00 ± 0.00	0.00 ± 0.00
	168	5.47 ± 0.03	3.00 ± 0.21	0.00 ± 0.00	0.00 ± 0.00	7.14 ± 0.12	3.50 ± 0.17	3.03 ± 0.13	0.00 ± 0.00
*E. coli*	0	3.69 ± 0.13	0.00 ± 0.00	0.00 ± 0.00	0.00 ± 0.00	4.53 ± 0.15	0.80 ±1.24	0.00 ± 0.00	0.00 ± 0.00
	24	4.74 ± 0.11	0.00 ± 0.00	0.00 ± 0.00	0.00 ± 0.00	5.61 ± 0.10	0.00 ± 0.00	0.00 ± 0.00	0.00 ± 0.00
	72	4.61 ± 0.06	0.00 ± 0.00	0.00 ± 0.00	0.00 ± 0.00	5.01 ± 0.05	0.00 ± 0.00	0.00 ± 0.00	0.00 ± 0.00
	168	2.91 ± 0.25	0.00 ± 0.00	0.00 ± 0.00	0.00 ± 0.00	5.29 ± 0.16	0.00 ± 0.00	0.00 ± 0.00	0.00 ± 0.00
Coliforms	0	4.26 ± 0.07	0.00 ± 0.00	0.00 ± 0.00	0.00 ± 0.00	3.61 ± 0.09	0.00 ± 0.00	0.00 ± 0.00	0.00 ± 0.00
	24	4.78 ± 0.06	0.00 ± 0.00	0.00 ± 0.00	0.00 ± 0.00	4.47 ± 0.13	0.00 ± 0.00	0.00 ± 0.00	0.00 ± 0.00
	72	3.76 ± 0.13	0.00 ± 0.00	0.00 ± 0.00	0.00 ± 0.00	4.24 ± 0.15	0.00 ± 0.00	0.00 ± 0.00	0.00 ± 0.00
	168	1.51 ± 1.66	0.00 ± 0.00	0.00 ± 0.00	0.00 ± 0.00	5.50 ± 0.14	0.00 ± 0.00	0.00 ± 0.00	0.00 ± 0.00
Other Gram-negative	0	5.68 ± 0.06	0.88 ± 1.37	0.39 ± 0.96	0.00 ± 0.00	5.02 ± 0.12	0.00 ± 0.00	0.00 ± 0.00	0.00 ± 0.00
	24	5.99 ± 0.14	0.00 ± 0.00	0.00 ± 0.00	0.00 ± 0.00	6.92 ± 0.07	2.36 ± 1.16	0.00 ± 0.00	0.00 ± 0.00
	72	7.16 ± 0.04	1.69 ± 1.32	0.00 ± 0.00	0.00 ± 0.00	5.52 ± 0.07	0.00 ± 0.00	0.00 ± 0.00	0.00 ± 0.00
	168	5.47 ± 0.03	3.00 ± 0.21	0.00 ± 0.00	0.00 ± 0.00	7.12 ± 0.13	3.50 ± 0.17	3.03 ± 0.13	0.00 ± 0.00
SSLO^j^	0	6.56 ± 0.14	6.42 ± 0.05	3.27 ± 0.26	0.00 ± 0.00	7.13 ± 0.09	6.47 ± 0.12	5.31 ± 0.04	3.79 ± 0.32
	24	7.31 ± 0.14	6.75 ± 0.02	5.13 ± 0.13	0.00 ± 0.00	7.66 ± 0.07	7.51 ± 0.12	7.19 ± 0.17	5.60 ± 0.06
	72	7.42 ± 0.09	6.82 ± 0.18	5.29 ± 0.10	1.55 ± 1.20	7.07 ± 0.18	6.93 ± 0.09	6.59 ± 0.13	5.75 ± 0.19
	168	6.16 ± 0.12	6.98 ± 0.07	5.08 ± 0.08	3.50 ± 0.12	7.07 ± 0.17	6.93 ± 0.15	6.71 ± 0.05	6.19 ± 0.11

^a^Standard deviation.

^b^Sample processed immediately (0 h), and after 24, 72, and 168 h of storage at 28°C.

^c^Anaerobically digested manure solids.

^d^Separated raw manure solids.

^e^0%: untreated control sample.

^f^10%: sample treated with 10% of lime-based conditioner.

^g^15%: sample treated with 15% of lime-based conditioner.

^h^20%: sample treated with 20% of lime-based conditioner.

^i^ TBC, total bacterial counts.

^j^Streptococci and streptococci-like organism.

**Table 5:** Within-timepoint models^a^ for microbiological counts of anaerobically digested manure solids and separated raw manure solids treated with lime-based conditioner.

**Bacterial group**	**Time-points (h)^b^**	**Term^c^**	**ADMS** ^**d**^				**SRMS** ^**e**^			
			**Estimate**	**SE^f^**	**Statistic**	***P*-value**	**Estimate**	**SE**	**Statistic**	***P*-value**
TBC^g^	0	Treatment	−0.133	0.005	−25.298	<0.001	−0.045	0.002	−20.570	<0.001
	24		−0.174	0.008	−21.867	<0.001	−0.155	0.005	−30.926	<0.001
	72		−0.177	0.008	−21.171	<0.001	−0.133	0.010	−12.873	<0.001
	168		−0.152	0.018	−8.597	<0.001	−0.134	0.011	−12.020	<0.001
Total Gram–negative	0	Treatment	−0.292	0.032	−9.022	<0.001	−0.270	0.028	−9.688	<0.001
	24		−0.311	0.036	−8.608	<0.001	−0.371	0.026	−14.302	<0.001
	72		−0.378	0.031	−12.029	<0.001	−0.291	0.034	−8.615	<0.001
	168		−0.299	0.018	−16.725	<0.001	−0.335	0.016	−21.553	<0.001
*E. coli*	0	Treatment	−0.190	0.022	−8.591	<0.001	−0.237	0.025	−9.486	<0.001
	24		−0.244	0.028	−8.605	<0.001	−0.289	0.034	−8.611	<0.001
	72		−0.237	0.028	−8.614	<0.001	−0.258	0.030	−8.614	<0.001
	168		−0.149	0.018	−8.468	<0.001	−0.272	0.032	−8.597	<0.001
Coliforms	0	Treatment	−0.219	0.025	−8.612	<0.001	−0.186	0.022	−8.604	<0.001
	24		−0.246	0.029	−8.613	<0.001	−0.230	0.027	−8.599	<0.001
	72		−0.194	0.023	−8.593	<0.001	−0.218	0.025	−8.589	<0.001
	168		−0.077	0.024	−3.287	0.003	−0.283	0.033	−8.603	<0.001
Other Gram-negative	0	Treatment	−0.291	0.032	−9.014	<0.001	−0.258	0.030	−8.606	<0.001
	24		−0.308	0.036	−8.605	<0.001	−0.370	0.026	−14.301	<0.001
	72		−0.378	0.031	−12.029	<0.001	−0.284	0.033	−8.614	<0.001
	168		−0.299	0.018	−16.719	<0.001	−0.334	0.016	−21.462	<0.001
SSLO^h^	0	Treatment	−0.318	0.038	−8.402	<0.001	−0.161	0.013	−11.978	<0.001
	24		−0.321	0.046	−7.061	<0.001	−0.089	0.014	−6.238	<0.001
	72		−0.268	0.036	−7.359	<0.001	−0.060	0.008	−7.220	<0.001
	168		−0.129	0.026	−5.061	<0.001	−0.040	0.005	−7.416	<0.001

^a^Within-timepoint model assessed the effects of incremental lime-based conditioner doses (0–20%) on DM and pH, at each timepoint (0, 24, 72, and 168 h).

^b^Sample processed immediately (0 h), and after 24, 72, and 168 h of storage at 28°C.

^c^Treatment: untreated control sample, and sample treated with 10, 15, and 20% of lime-based conditioner.

^d^Anaerobically digested manure solids.

^e^Separated raw manure solids.

^f^Standard error.

^g^Total bacterial count.

^h^Streptococci and streptococci-like organism.

**Table 6:** Across-timepoint models^a^ for microbiological counts of anaerobically digested manure solids and separated raw manure solids treated with lime-based conditioner.

**Bacterial group**	**Term^b^**	**ADMS** ^**c**^				**SRMS** ^**d**^			
		**Estimate**	**SE^e^**	**Statistic**	***P*-value**	**Estimate**	**SE**	**Statistic**	***P*-value**
TBC^f^	24 h	0.146	0.119	1.229	0.222	−0.295	0.125	−2.366	0.020
	72 h	0.745	0.119	6.282	<0.001	0.139	0.125	1.118	0.266
	168 h	0.913	0.119	7.693	<0.001	0.360	0.125	2.892	0.004
	Treatment	−0.159	0.006	−28.076	<0.001	−0.117	0.006	−19.637	<0.001
Total Gram-negative	24 h	−0.233	0.320	−0.729	0.468	0.836	0.287	2.915	0.004
	72 h	0.471	0.320	1.472	0.144	−0.078	0.287	−0.271	0.787
	168 h	0.375	0.320	1.172	0.244	1.927	0.287	6.722	<0.001
	Treatment	−0.320	0.015	−20.91	<0.001	−0.317	0.014	−23.107	<0.001
*E. coli*	24 h	0.261	0.263	0.993	0.324	0.071	0.313	0.226	0.822
	72 h	0.229	0.263	0.868	0.387	−0.080	0.313	−0.256	0.799
	168 h	−0.197	0.263	−0.747	0.457	−0.011	0.313	−0.034	0.973
	Treatment	−0.205	0.013	−16.278	<0.001	−0.264	0.015	−17.625	<0.001
Coliforms	24 h	0.129	0.294	0.440	0.661	0.215	0.288	0.748	0.457
	72 h	−0.125	0.294	−0.425	0.672	0.158	0.288	0.551	0.583
	168 h	−0.690	0.294	−2.342	0.021	0.471	0.288	1.639	0.105
	Treatment	−0.184	0.014	−13.078	<0.001	−0.229	0.014	−16.666	<0.001
Other Gram–negative	24 h	−0.241	0.319	−0.755	0.452	1.065	0.293	3.640	0.001
	72 h	0.476	0.319	1.492	0.139	0.123	0.293	0.421	0.675
	168 h	0.380	0.319	1.191	0.237	2.157	0.293	7.374	<0.001
	Treatment	−0.319	0.015	−20.907	<0.001	−0.311	0.014	−22.269	<0.001
SSLO^g^	24 h	0.502	0.429	1.170	0.245	1.314	0.151	8.684	<0.001
	72 h	1.208	0.415	2.911	0.004	0.906	0.151	5.989	<0.001
	168 h	1.368	0.415	3.298	0.001	1.047	0.151	6.921	<0.001
	Treatment	−0.259	0.020	−13.055	<0.001	−0.088	0.007	−12.118	<0.001

^a^For the across-timepoint analysis, all data (for ADMS and SRMS separately) were analyzed jointly, considering the effects of incremental lime-based conditioner doses (0–20%) on DM and pH, and including in the model the effect of the number of hours of lime-based conditioner application.

^b^Sample processed after 24, 72, and 168 h of storage at 28°C, respectively; Treatment: untreated control sample, and sample treated with 10, 15, and 20% of lime-based conditioner.

^c^Anaerobically digested manure solids.

^d^Separated raw manure solids.

^e^Standard error.

^f^Total bacterial count.

^g^Streptococci and streptococci-like organism.

Regarding the total Gram-negative counts, untreated samples had higher counts compared to treated samples (Table 4). Interestingly, samples treated with 20% LBC showed no Gram-negative bacterial growth throughout the study period (Table 4). A linear decrease in total Gram-negative counts with LBC addition was observed for both ADMS and SRMS at all evaluated time-points (*P* < 0.001; Table 5). While no difference in Gram-negative counts was observed for ADMS over time (*P* ≥ 0.144), an increase in Gram-negative counts was observed for SDMS after 24 h (*P* = 0.004) and 168 h (*P* < 0.001) of sample incubation. The addition of LBC had a negative effect on Gram-negative counts, reducing counts by 0.32 log_10_ cfu/g for both ADMS (*P* < 0.001) and SRMS for each % unit of LBC inclusion (*P* < 0.001; Table 6).

*E. coli* was not isolated from ADMS and SRMS treated with LBC throughout the study duration, except for fresh SRMS treated with 10% of LBC (0.80 ± 1.24 log_10_ cfu/g; Table 4). No differences were observed in *E. coli* counts over time (*P* ≥ 0.32; Table 6). *E. coli* counts reduced by 0.21 log_10_ cfu/g for ADMS (*P* < 0.001) and 0.26 log_10_ cfu/g for SRMS per % unit of LBC added (*P* < 0.001; Table 6).

Similar to *E. coli* counts, no coliform growth was observed during the study for LBC-treated ADMS and SRMS (Table 4). Except for ADMS evaluated at 168 h (*P* = 0.021), no differences were observed in coliform counts over time for ADMS and SRMS (*P* ≥ 0.105; Table 6). For each % unit of LBC added, coliform counts were reduced by 0.18 log_10_ cfu/g for ADMS (*P* < 0.001) and 0.23 log_10_ cfu/g for SRMS (*P* < 0.001; Table 6).

A decrease in the counts of other Gram-negative bacteria according to the LBC concentration was observed for both ADMS and SRMS (Table 4). Regardless of the evaluation time, untreated samples had higher other Gram-negative counts compared to those treated with LBC. No other Gram-negative growth was observed throughout the study for both ADMS and SRMS treated with 20% LBC (Table 4). A decrease in other Gram-negative bacteria counts with LBC addition was observed for both ADMS and SRMS (*P* ≤ 0.001; Table 5). No difference in other Gram-negative counts over time was observed for ADMS (*P* ≥ 0.139), while an increase in other Gram-negative counts was observed for SDMS after 24 h (*P* = 0.001) and 168 h (*P* < 0.001) of sample incubation. Each % unit of LBC added decreased other Gram-negative bacterial counts by 0.32 log_10_ cfu/g for ADMS (*P* < 0.001) and 0.31 log_10_ cfu/g for SRMS (*P* < 0.001; Table 6).

LBC treatment of ADMS and SRMS was also effective in reducing SSLO counts, with samples at 20% LBC inclusion having the lowest SSLO count compared to untreated samples or those treated with lower LBC concentrations (Table 4). A linear decrease in SSLO count was observed according to increasing LBC concentration (*P* < 0.001; Table 5). Over time, a consistent increasing pattern in SSLO count was observed for both substrates (*P* ≤ 0.004; Table 6), except for ADMS after 24 h (*P* = 0.245). For each % unit of LBC inclusion, SSLO counts were reduced by 0.26 log_10_ cfu/g for ADMS (*P* < 0.001) and by 0.09 log_10_ cfu/g for SRMS, respectively (*P* < 0.001; Table 6).

## 4 Discussion

Controlling pathogen growth in bedding substrates is critical to preventing intramammary infections, especially for organic bedding materials such as RMS, which has been reported to support higher levels of environmental mastitis pathogens (6, 22). To our knowledge, this is the first study that describes the effect of different concentrations of a commercial LBC on the physical-chemical and microbiological characteristics of RMS. Our findings underscore the potential of LBC in controlling environmental bacteria and optimizing the physical-chemical characteristics of bedding materials to improve cow health and welfare.

In our study, bacterial counts on untreated substrates remained high or increased over time. However, when LBC was applied, we observed a decrease in TBC, Gram-negative bacteria, coliforms, *E. coli*, and SSLO counts with increasing LBC concentration, suggesting the potential of LBC to control pathogen proliferation. Consistent with our findings, other alkaline conditioners and hydrated lime have been shown to effectively inhibit bacteria growth in RMS (11). It has been reported that the antibacterial activity of bedding conditioners is related to pH (11, 23). In our study, the addition of LBC increased the pH to the alkaline range. As the bedding pH increased a decrease in bacterial counts was observed, and this was more pronounced as higher concentrations of LBC were added. Similarly, Hogan et al. (11) reported that alkaline-based conditioners were more effective in controlling bacterial populations in RMS, considering this substrate had a near-neutral pH, effectively inhibiting bacteria in RMS for 1 day.

In addition, after reaching the highest pH level with the inclusion of LBC, we observed a decreasing trend in pH over time for both substrates (ADMS and SRMS). As the pH of the substrates decreased over time, the antibacterial effects of the LBC decreased, but the bacterial recovery appeared slower with higher LBC concentrations. In previous reports, the use of disinfectants (e.g., acid and alkaline conditioners, and hydrated lime) initially reduced bacterial counts, but the antimicrobial activity had diminished by 2 days after application (11, 16). In our study, the antimicrobial activity of the LBC started to diminish by 24 h. Higher concentrations of LBC may extend the antibacterial effect in bedding materials, which may be of practical interest for bedding management on farms.

Several studies have reported an association between the counts of mastitis pathogens in bedding materials and on the teat ends (24, 25), and the intensity of udder contamination has been associated with an increased risk of mastitis (26). Controlling bedding moisture may be one way to reduce the risk of mastitis (27, 28). In our study, a linear increase in DM content as a function of LBC concentration was observed for both ADMS and SRMS. This suggests that in addition to controlling microbial growth by altering the pH, the inclusion of LBC also prevents bacterial growth by increasing the DM content of the bedding material. An increase in DM content in RMS treated with alkaline conditioner, acid conditioner, or hydrated lime was also observed by Hogan et al. (11). Robles et al. (29) highlighted the association of a higher percentage of DM in the bedding with reduced bacterial counts, as well as with higher bulk tank milk quality.

Finally, the results of our study must be interpreted with caution due to its *in vitro* nature. *In vitro* studies are conducted under controlled conditions; excluding the presence of feces, urine, and other contaminants that might affect the results. Several factors limit the antimicrobial effect of bedding conditioners to 24–48 h, such as contamination of bedding by cows entering the facility, removal of the bedding and conditioner when cows exit the stalls, and buffering capacity of the bedding conditioner over time (16). Field studies should be performed to validate our findings and the safety of LBC concentrations concerning cow health, considering previous studies described that hydrated lime applied to a free-stall mattress every 48 h caused mild ulceration and scaling on the cow's legs and udder (9). To our knowledge, there is no evidence (field studies) to support the safe use of bedding conditioners for dairy cows. Therefore, our study can serve as a great starting point for future field studies to evaluate the effect of different LBC concentrations on bedding characteristics, mastitis risk and cow safety.

## 5 Conclusion

A linear relationship between DM content and pH was observed with increasing LBC concentration. Both bedding substrates (ARMS and SRMS) with no inclusion of LBC presented higher bacterial counts, which tended to remain high or increase over time compared to the LBC-treated samples. On the other hand, when LBC was applied to ARMS and SRMS, a linear decrease in TBC, other Gram-negative bacteria, coliforms, *E. coli*, and SSLO counts was observed. This reduction in bacterial counts can be attributed to increased bedding pH and DM. The pH reduction and the bacterial recovery were slower over time with higher concentrations of LBC.

## Data availability statement

The raw data supporting the conclusions of this article will be made available by the authors, without undue reservation.

## Author contributions

GF: Writing – review & editing, Writing – original draft, Methodology, Investigation, Data curation, Conceptualization. SF: Visualization, Validation, Writing – review & editing, Writing – original draft, Methodology, Investigation, Data curation, Conceptualization. VM: Validation, Methodology, Investigation, Conceptualization, Writing – review & editing, Writing – original draft. FB: Writing – review & editing, Writing – original draft, Formal analysis, Data curation. NR: Writing – review & editing, Writing – original draft, Investigation. DH: Writing – review & editing, Writing – original draft, Investigation. CG: Writing – review & editing, Writing – original draft, Investigation. LM: Methodology, Formal analysis, Writing – review & editing. GG: Writing – review & editing, Writing – original draft. LL: Writing – review & editing, Writing – original draft. MV: Writing – review & editing, Writing – original draft. PM: Writing – review & editing, Writing – original draft, Supervision, Project administration, Methodology, Funding acquisition. MA: Writing – review & editing, Writing – original draft, Methodology. VB: Writing – review & editing, Writing – original draft, Project administration, Methodology, Investigation, Formal analysis.
